# A Novel Inflammatory Response-Related Gene Signature Predicts Immune Status and Prognosis of Breast Cancer

**DOI:** 10.1155/2022/5468858

**Published:** 2022-11-23

**Authors:** Ruijun Zhao, Chaoyu Xie, Yu Gong, Songzhi Wei, Mei Yuan, Jinfeng Gan, Wenyan Chen

**Affiliations:** ^1^Department of Breast Surgery, The Third Hospital of Nanchang, Nanchang, China; ^2^Department of Pathology, School of Medicine, Jinan University, Guangzhou, China; ^3^Department of Medical Oncology, The Third Hospital of Nanchang, Nanchang, China; ^4^Department of General Surgery, Xinfeng People's Hospital, Ganzhou, China; ^5^Guangxi Key Laboratory of Tumor Immunology and Microenvironmental Regulation, Guilin Medical University, Guilin, China

## Abstract

**Purpose:**

Breast cancer is the most common type of cancer and the leading cause of cancer-related death in women worldwide. In this study, we aimed to construct an inflammatory response-related gene model for predicting the immune status and prognosis of breast cancer patients.

**Methods:**

We obtained the inflammatory response-related genes from the Molecular Signatures Database. Furthermore, we used univariate Cox regression analysis, the least absolute shrinkage and selection operator (LASSO) regression analysis, and multivariate Cox regression to construct an inflammatory response-related gene signature (IRGS) model based on dataset obtained from The Cancer Genome Atlas (TCGA). Patients were consequently categorized into high-risk and low-risk groups. Kaplan–Meier analysis was used to compare the overall survival (OS) of high-risk and low-risk groups. Following that, we validated the model using a dataset (GSE96058) acquired from Gene Expression Omnibus (GEO) database. Univariate and multivariate Cox analyses were used to determine the independent prognostic value of the IRGS in the TCGA and GSE96058 cohorts. A nomogram was constructed to predict the OS in the TCGA cohort. Further, we used Gene Set Enrichment Analysis (GSEA), CIBERSORT, and single-sample Gene Set Enrichment Analysis (ssGSEA) to evaluate the associations of IRGS with immune-associated pathways and immune infiltration. Finally, the relationship between the expression of the signature genes and drug sensitivity was conducted using Pearson correlation analysis.

**Results:**

We established an IRGS to stratify breast cancer patients into the low-risk and high-risk groups. In both the training and validation sets, patients in the high-risk group had significantly shorter OS than those in the low-risk group. The risk score was significantly correlated with the clinical characteristics and could be used as a tool to predict the prognosis of breast cancer. Moreover, we found that the IRGS risk score was an independent predictor of OS in breast cancer patients, and a nomogram model based on IRGS risk score and other clinical factors could effectively predict the prognosis of breast cancer patients. Furthermore, the IRGS risk score was correlated with immune characteristics and was inversely associated with the abundance of immune cell infiltration. Patients with a low IRGS risk score had higher expression levels of immune checkpoint genes, suggesting that IRGS can be used as a potential indicator for immunotherapy. Finally, we found that the expression levels of prognostic genes were significantly correlated with tumor cell sensitivity to chemotherapeutic drugs.

**Conclusion:**

Overall, these findings suggest that the IRGS can be used to predict the prognosis and immune status of breast cancer patients and provide new therapeutic targets for the treatment of these patients.

## 1. Introduction

Breast cancer is the most common type of cancer in women around the world [1]. In 2021, breast cancer accounted for 30% of newly diagnosed cancer cases and 15% of cancer-related deaths in women [2]. Surgical resection, radiotherapy, adjuvant chemotherapy, endocrine therapy, molecular targeted therapy, and immunology therapy are commonly used for the treatment of breast cancer [3]. Although these comprehensive treatment methods have a curative effect, tumor recurrence and metastasis frequently cause poor prognosis in breast cancer patients [3]. Breast cancer is a highly heterogeneous disease with a complex etiology, and the systemic inflammatory response is critical in the development and progression of the disease [4]. Thus, there is an urgent need to find effective and valuable inflammatory response-related biomarkers to improve prognosis prediction and clinical outcome of patients with breast cancer.

Inflammation is a hallmark of cancer, and it plays an important role in cancer development and progression [5, 6]. A growing body of evidence suggests that cancer-related inflammatory responses regulate immune cell infiltration and immune response, affecting tumor proliferation, invasion, and metastasis [7]. Inflammation is mediated by a variety of cytokines, chemokines, and hormones that help to regulate a wide range of processes involved in breast cancer development [4]. Furthermore, inflammatory cytokines and other inflammatory mediators in the tumor microenvironment (TME) can influence immune function, tumor growth, differentiation, invasion, and metastasis [8]. It has been reported that elevated levels of inflammation-related markers CRP, TNF, IL-6, and IL-8 are associated with poor prognosis in breast cancer patients [9]. A previous study has shown that IL-6 expression is correlated with tumor stage, lymph node metastasis, and a poor prognosis in breast cancer [10]. IL-1*β* is a proinflammatory cytokine that is associated with a poor prognosis in breast cancer due to its ability to stimulate NF-kappaB-driven gene transcription [11]. IL-8, a member of the CXC chemokine family of inflammation-related chemokines, is significantly overexpressed in human breast cancer patients and is associated with a poor prognosis [12]. TNF*α* is a proinflammatory cytokine that stimulates cell proliferation by increasing cyclin D1 transcription in ER-positive breast cancer [13]. Previous studies revealed that increased CRP levels are associated with an increased risk of breast cancer [14]. Several studies on inflammatory responses revealed that adjuvant chemotherapy causes a severe systemic inflammatory response and a weak adaptive immune response, which promotes breast cancer progression and poor prognosis [15]. To date, increasing evidence has confirmed that some inflammation-related markers are closely associated with the prognosis of breast cancer. However, there is no report on the inflammatory response-related gene signature (IRGS) as a predictor of breast cancer.

In this study, we constructed a prognostic IRGS for breast cancer using a The Cancer Genome Atlas (TCGA) cohort and validated it in the Gene Expression Omnibus (GEO) dataset. The Human Protein Atlas database, which includes immunohistochemical (IHC) images, was used to assess inflammation-related gene expression. Then, using the risk score, we created a nomogram for predicting 1-, 3-, and 5-year overall survival (OS) of breast cancer patients in the TCGA cohort. In addition, we explored the relationship between the risk score model and tumor immune status. Finally, we investigated the relationship between the expression of prognostic genes and cancer cell chemoresistance.

## 2. Methods and Materials

### 2.1. Data Acquisition

RNA expression data and corresponding clinical information of breast cancer patients were systematically obtained from the TCGA and GEO databases (GSE96058 cohort) [16]. The TCGA cohort contained 1222 tissues, including 113 normal tissues and 1109 tumor tissues and was used as the training cohort. The GSE96058 cohort, which contained 3273 samples, was used as the external validation cohort. The RNA sequencing data downloaded from the TCGA database were transformed into the same format as the GSE96058 cohort (log2 (FPKM + 0.1)).

### 2.2. Identification of Differentially Expressed Inflammatory Response-Related Genes

Inflammatory response-related genes were collected from the gene sets “GOBP_INFLAMMATORY_RESPONSE” and “HALLMARK_INFLAMMATORY_RESPONSE” in the Molecular Signatures Database (MSigDB) (https://www.gsea-msigdb.org/), which are shown in Supplementary Table S1 and Supplementary Table S2. In the TCGA cohort, differential gene expression analysis for inflammatory response-related genes was performed using the Wilcox test. Genes with |log2 fold change (FC)| > 1 and a false discovery rate (FDR) < 0.05 were considered as differential expression genes (DEGs). The DEGs were visualized in the form of a heatmap using the R package “pheatmap.”

### 2.3. Development and Validation of a Prognostic IRGS

To better understand the relationship between DEGs and patient survival, the R package “survival” was used to perform a univariate Cox analysis based on the DEGs. The IRGS was then developed using the least absolute shrinkage and selection operator (LASSO) regression (R package “glmnet”) and multivariate Cox regression analysis. The risk scores of the patients were calculated as follows: risk score = *β*_1_*x*_1_ + *β*_2_*x*_2_ + ... + *β*_i_*x*_i_. In this formula, *x*_i_ represented the expression level of signature genes and *β*_i_ represented the corresponding coefficient based on the results of multivariate Cox regression analysis.

The breast cancer patients in the TCGA cohort were divided into low- and high-risk groups according to the median risk score, and patients in the GSE96058 cohort were divided into low- and high-risk groups based on the same cutoff value. Through the R packages “survival” and “survminer,” Kaplan–Meier analysis was employed to compare OS between the low- and high-risk groups. Furthermore, time-dependent receiver operating characteristic (ROC) curves for 1-, 3-, and 5-year OS were plotted using the R package “timeROC.” IHC staining of prognostic signature genes was observed using the Human Protein Atlas database (https://www.proteinatlas.org/) [17].

For subgroup analysis, patients in the TCGA and GSE96058 cohorts were divided into two groups based on the corresponding clinical parameters, and Kaplan–Meier analysis and correlation analysis were performed.

### 2.4. Construction and Evaluation of a Nomogram

To verify whether the IRGS was independent of other clinical variables in predicting OS in breast cancer patients, univariate and multivariate Cox regression analyses were conducted by the “survival” R package in both the TCGA and GSE96058 cohorts.

The independent clinical prognostic factors that were identified by multivariate Cox regression analysis in the TCGA cohort were included in a nomogram to predict the OS of 1-, 3-, and 5-year using the R package “rms.” The concordance index (C-index) was calculated to quantify the predictive accuracy of the nomogram. The calibration curves were plotted to compare the predicted and actual OS rates.

### 2.5. Gene Set Enrichment and Immune Status Analyses

Gene Set Enrichment Analysis (GSEA) was conducted to examine the biological difference between high- and low-risk groups. The analysis was carried out with the R package “clusterProfiler,” and *P* value <0.05 was considered to be statistically significant.

Next, multiple methods, including ESTIMATE, CIBERSORT, and single-sample GSEA (ssGSEA), were utilized to characterize the difference in immune status between the high- and low-risk groups. The ESTIMATE algorithm was used to calculate stromal scores, immune scores, and estimate scores for each sample by using the R package “estimate.” The CIBERSORT algorithm was used to estimate the proportion of 22 immune cells in each sample by using the R software. The ssGSEA was used to estimate the proportion of 28 immune cells in each sample by using the R package “GSVA.”

### 2.6. Chemotherapy Sensitivity Analysis

To investigate whether the signature genes were related to the drug sensitivity of chemotherapy, we used the CellMiner website (https://discover.nci.nih.gov/cellminer) to access the NCI-60 database, which contained 60 different cancer cell lines from nine different types of tumors; then, Pearson correlation analysis was employed to assess the relationship between signature genes and efficacy of chemotherapy drugs approved by the FDA or currently used in clinical trials.

### 2.7. Statistical Analysis

R software (version 4.1.0) was used for all statistical analyses and visualization in this study. The Wilcoxon test was used to compare DEGs between tumor and normal tissues. To assess the relationship between IRGS and prognosis, univariate and multivariate Cox proportional hazard regression analyses were performed using the R packages “survival” and “survminer.” The multivariate model was used to screen the genes with prognostic significance (*P* value <0.10) [18]. The Kaplan–Meier method was used to calculate the survival analyses. The R package “timeROC” was used to assess the predictive ability of the IRGS. Statistical significance was defined as a *P* value less than 0.05.

## 3. Result

### 3.1. Construction of a Prognostic IRGS for Breast Cancer Using the TCGA Cohort

**Figure 1:**
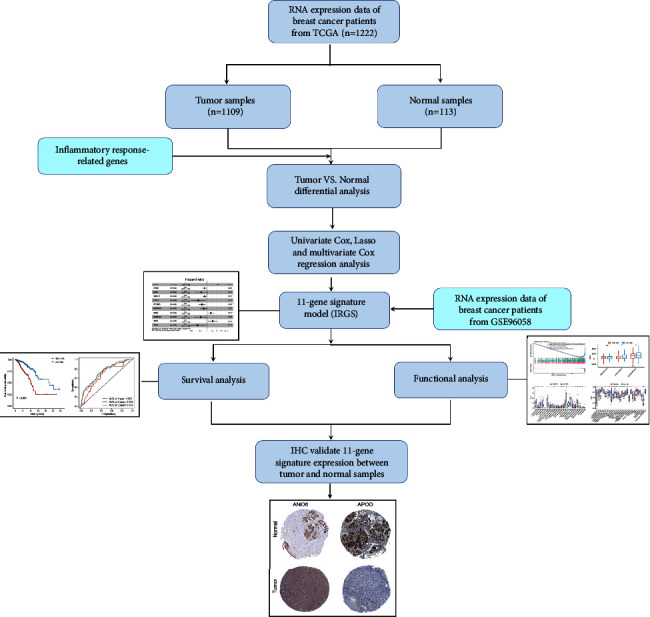
Flow chart of sample collection and analysis.

The flow chart of this study is shown in Figure 1. We collected 1109 breast cancer specimens and 113 normal specimens from the TCGA database for this study. Another cohort of 3273 breast cancer specimens was obtained from the GEO database (GSE96058). Patients who did not have follow-up data were excluded, resulting in a cohort of 1069 cases in the TCGA dataset and 3069 cases in the GSE96058 dataset.

**Figure 2:**
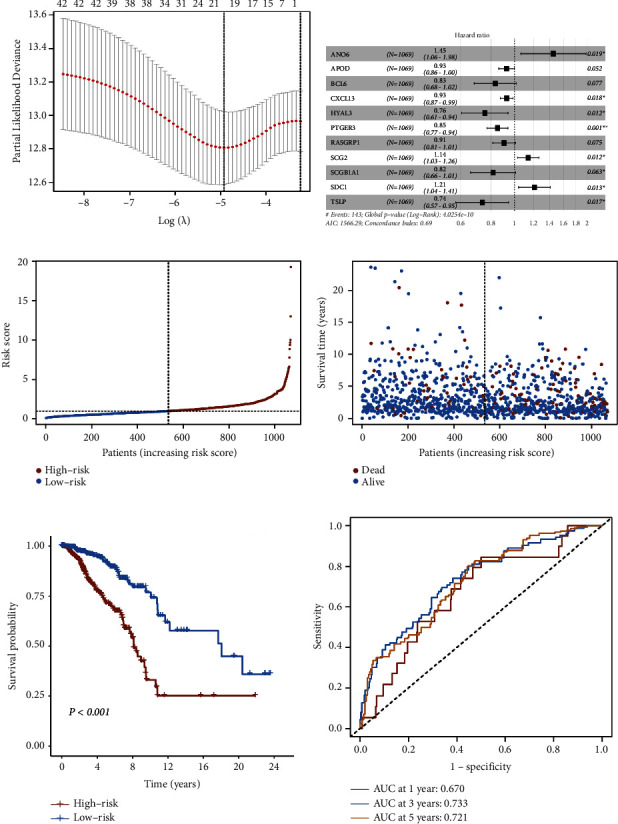
Construction of the IRGS using the TCGA cohort. The prognostic signature was developed by LASSO Cox regression analysis (a) and multivariate analysis (b) of candidate genes that were associated with OS of breast cancer patients in the TCGA cohort. (c) Breast cancer patients in the TCGA cohort were separated into the high-risk and low-risk groups with the median value of risk score. (d) The survival status and risk score distribution in the TCGA cohort. (e) Kaplan–Meier curves of OS between the high-risk and low-risk groups in the TCGA cohort. (f) ROC curves of the risk score to predict the 1-, 3-, and 5-year OS in the TCGA cohort.

Since inflammatory response plays a crucial role in the development and progression of breast cancer, we first examined the expression heterogeneity of inflammatory response-related genes in the tumor and normal tissues. As a result, 243 inflammatory response-related genes were found to be differentially expressed in the TCGA cohort (Supplementary Figure 1A, FDR <0.05, |log2 fold change (FC)| > 1). To comprehensively understand the prognostic value of the inflammatory response-related genes in breast cancer, a univariate Cox proportional hazards regression analysis was employed, and a total of 45 genes were found to be associated with the OS (*P* < 0.05) (Supplementary Figure 1B). Next, we performed a LASSO Cox regression analysis on these factors, and finally, we chose 19 genes that appeared to be stable factors based on the optimal lambda (*λ*) value (Figure 2(a) and Supplementary Figure 1C). Ultimately, all factors obtained from the LASSO Cox regression analysis were included in the multivariate Cox regression analysis (Figure 2(b)). Based on the results of multivariate Cox regression analysis, 11 genes were selected to establish the IRGS, and a risk score formula was developed: risk score = ANO6 expression × (0.3713) + APOD expression × (−0.0747) + BCL6 expression × (−0.1823) + CXCL13 expression × (−0.0732) + HYAL3 expression × (−0.2807) + PTGER3 expression × (−0.1609) + RASGRP1 expression × (−0.0981) + SCG2 expression × (0.1316) + SCGB1A1 expression × (−0.2036) + SDC1 expression × (0.1917) + TSLP expression × (−0.3063) (Supplementary Table S3).

Using the above risk score formula, the corresponding IRGS risk score for each patient in the TCGA cohort was calculated. Furthermore, the median risk score was used as the cutoff value to divide the patients into the high-risk (*n* = 535) and low-risk (*n* = 534) groups (Figure 2(c)). The distribution of IRGS risk score and patient survival status revealed that patients with a high-risk score had a higher probability of death than those with a low-risk score (Figure 2(d)). Consistently, patients in the high-risk group had significantly shorter OS than those in the low-risk group (*P* < 0.001, Figure 2(e)). Following that, to evaluate the prognostic performance of the IRGS, a time-dependent ROC analysis was performed, and the area under the curve (AUC) for 1-, 3-, and 5-year OS of this prognostic signature was 0.670, 0.733, and 0.721, respectively (Figure 2(f)). These findings indicate that the risk score derived from the IRGS has a strong prognostic value and high accuracy for predicting the OS of breast cancer patients.

### 3.2. Validation of the Prognostic Value of the IRGS Risk Score in the GSE96058 Cohort

**Figure 3:**
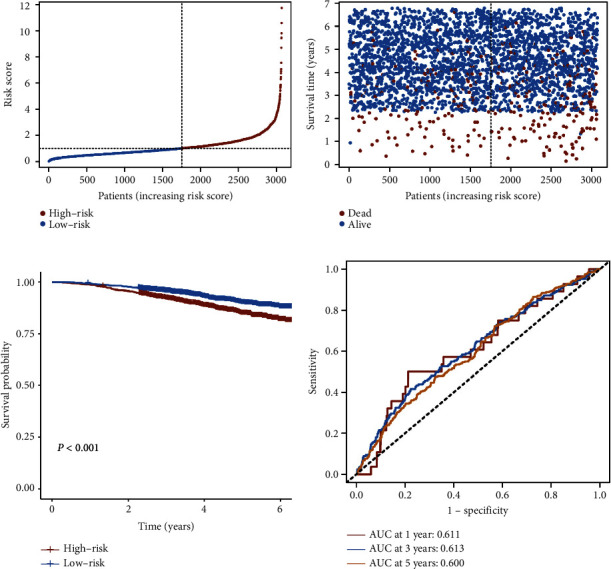
Validation of the prognostic value of the IRGS in the GSE96058 cohort. (a) Breast cancer patients in the GSE96058 cohort were separated into the high-risk and low-risk groups with the median value of the risk score in the TCGA cohort. (b) The survival status and risk score distribution in the GSE96058 cohort. (c) Kaplan–Meier curves of OS between the high-risk and low-risk groups in the GSE96058 cohort. (d) ROC curves of the risk score to predict the 1-, 3-, and 5-year OS in the GSE96058 cohort.

**Figure 4:**
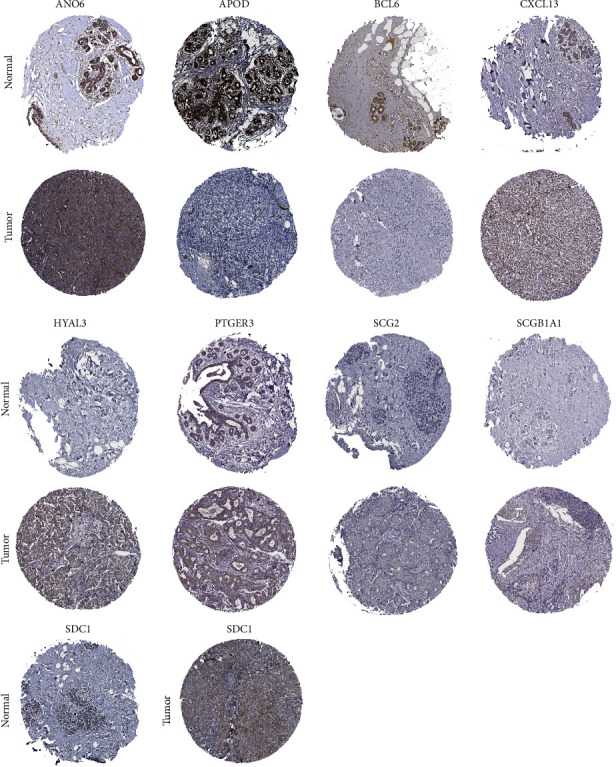
IHC staining of inflammatory response-related genes (ANO6, APOD, BCL6, CXCL13, HYAL3, PTGER3, SCG2, SCGB1A1, and SDC1) expression in breast cancer and normal tissues in the Human Protein Atlas database. The IHC data for RASGRP 1 and TSLP were not available in the Human Protein Atlas database.

To evaluate the robustness of the IRGS, we defined GSE96058 dataset (*n* = 3069) as an external validation cohort to test the prognostic power of this IRGS. The same risk score formula used in the TCGA training cohort was used to generate risk scores for each patient in the validation cohort (Figure 3(a)). According to the distribution of the IRGS risk scores and survival status of patients, patients at high risk had a higher probability of death than those at low-risk (Figure 3(b)). In the validation cohort, patients were divided into the high-risk (*n* = 1315) and low-risk (*n* = 1754) groups based on the same cutoff value as in the TCGA cohort. Patients with a low-risk score had substantially longer OS than those with a high-risk score (*P* < 0.001) (Figure 3(c)). The AUC for 1-, 3-, and 5-year OS of IRGS was 0.611, 0.613, and 0.600, respectively (Figure 3(d)). These findings were consistent with the findings from the TCGA cohort and supported the prognostic value of this IRGS. To further validate the findings, the protein expression of genes from the IRGS was measured using data from the Human Protein Atlas database, which includes IHC images of cancer and normal tissues (Figure 4).

### 3.3. The Correlation of IRGS Risk Score with Clinical Characteristics in Breast Cancer

**Figure 5:**
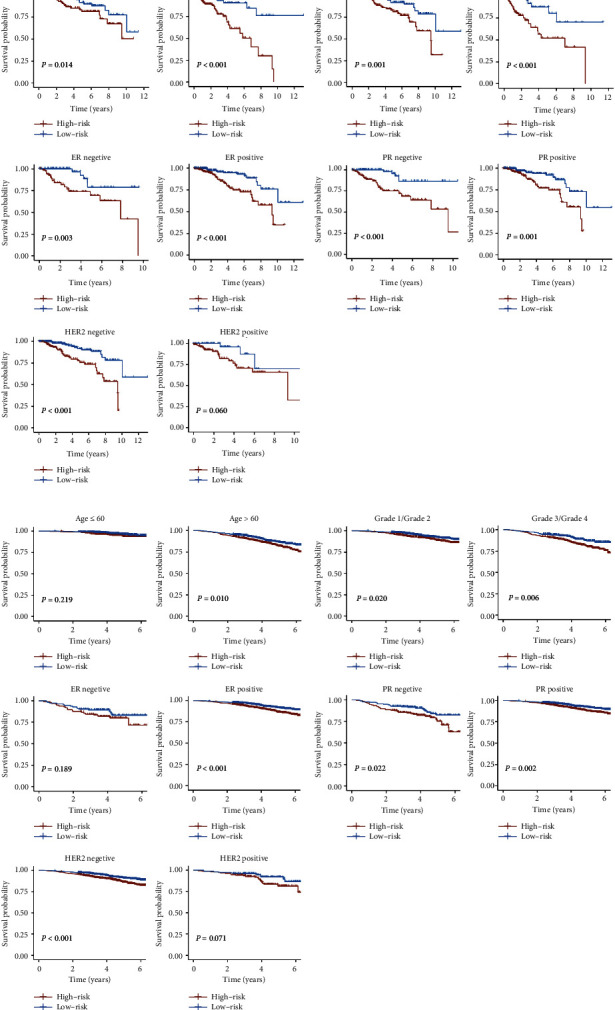
Kaplan–Meier curves analysis of patients with different clinical characteristics. The prognostic value of IRGS in different subgroups in the TCGA cohort (a) and the GSE96058 cohort (b).

To determine whether the IRGS risk score was correlated with clinical characteristics, we analyzed the differences in the risk scores among the various subgroups stratified by clinical characteristics. In the TCGA cohort, the risk scores were found to be closely related to age, PR, and HER2 status (Supplementary Figure 2A). The risk score in the stage and ER status subgroups, however, was not statistically different (Supplementary Figure 2A). In the GSE96058 cohort, except for the ER status, the risk score was significantly associated with age, grade, PR, and HER2 status (Supplementary Figure 2B). Further, we explored the prognostic value of IRGS in different subgroups based on clinical characteristics. In the TCGA cohort, a high-risk score was associated with a worse prognosis than a low-risk score in all subgroups except the HER2-positive subgroup (Figure 5(a)). In the GSE96058 cohort, except for the age ≤ 60, ER-negative, and HER2-positive subgroups, the risk score can effectively predict the OS of patients with various clinicopathological features (Figure 5(b)). Taken together, the results suggest that the risk score is closely associated with the clinical characteristics of breast cancer and can be used to predict the prognosis of breast cancer patients.

### 3.4. Independent Prognostic Value of the IRGS in the TCGA and GSE96058 Cohorts

**Figure 6:**
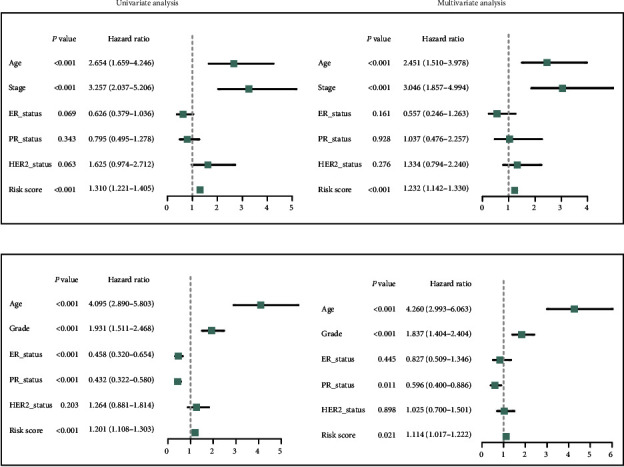
Univariate and multivariate Cox regression analyses of the IRGS on OS in the TCGA cohort (a) and the GSE96058 cohort (b).

To further determine whether the IRGS can be used as an independent prognostic factor for OS, the risk score and clinical factors were integrated into univariate and multivariate analyses. The risk score was significantly correlated with OS in both TCGA (hazard ratio (HR) = 1.232, 95% CI = 1.142–1.330, *P* < 0.001; Figure 6(a)) and GSE96058 (HR = 1.114, 95% CI = 1.017–1.222, *P* = 0.021; Figure 6(b)) cohorts after adjusting for other clinical characteristics indicating that risk score was an independent indicator for OS of breast cancer patients. Together, these results strongly demonstrate that the IRGS risk score can independently predict the OS of breast cancer patients.

### 3.5. Construction of a Prognostic Nomogram to Predict the OS of Breast Cancer Patients

**Figure 7:**
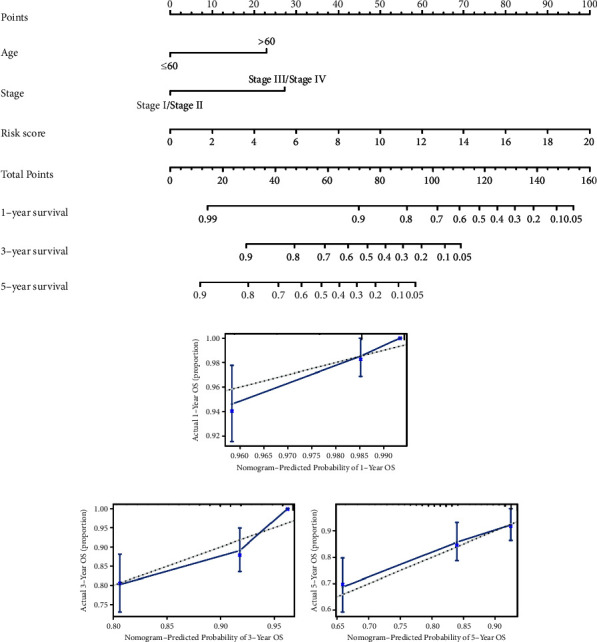
Nomogram to predict prognostic probabilities in the TCGA cohort. (a) Nomogram for predicting 1-, 3-, and 5-year OS of breast cancer patients. Draw vertical lines upwards to determine the points from each clinical variable. The points of each clinical variable were then added to determine the total points. A vertical line is drawn down from the Total Points axis to obtain the predicted OS probability of breast cancer patients. (b–d) Calibration curves of 1-, 3-, and 5-year OS of breast cancer patients.

A nomogram was constructed in the TCGA cohort to quantitatively predict the survival probability of breast cancer patients. The risk score, age, and tumor stage were integrated into the nomogram to predict the OS of breast cancer patients (Figure 7(a)). Furthermore, the predictive performance of the nomogram was evaluated by computing the C-index and generating the calibration curve of the model for 1-, 3-, and 5-year survival rates. As a result, the C-index of the nomogram was 0.800, and the calibration curves suggested that the predicted survival rate was similar to the actual survival rate of 1-, 3-, and 5-year (Figures 7(b)–7(d)), indicating the nomogram had the great predictive ability.

### 3.6. Identifying Biological Function and Immune Cells Related to the IRGS in Breast Cancer

**Figure 8:**
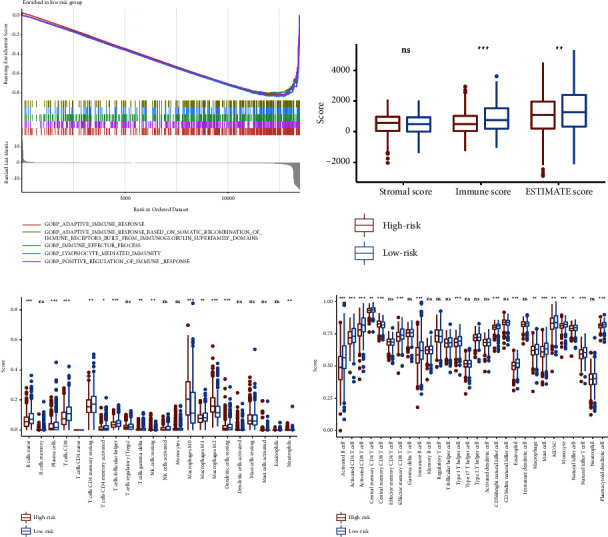
Identifying the biological functions and immune cells related to the IRGS in the TCGA cohort. (a) GSEA plot depicting immune-related gene sets enriched in patients with low risk. (b) Distribution of stromal score, immune score, and estimate score in the high-risk and low-risk groups in the TCGA cohort. (c, d) Comparison of the immune cell status between the high-risk and low-risk groups in the TCGA cohort by the CIBERSORT algorithm (c) and ssGSEA (d).

To determine whether the IRGS risk score can predict immune characteristics, we evaluated the relationship between the risk score and the immune status of breast cancer patients. Using GSEA, we found that the immune-related gene sets were enriched in patients with low risk in the TCGA cohort (Figure 8(a), *P* < 0.05 for all). The findings of the ESTIMATE algorithm revealed that samples from the low-risk group had significantly higher immune scores than those from the high-risk group, suggesting that the level of immune cell infiltration increased in the low-risk group (Figure 8(b)). To further investigate the relationship between the risk score and immune cell subpopulations, CIBERSORT and ssGSEA were employed. The immune cell infiltration analysis showed a dramatically inverse correlation between the IRGS risk score and the abundance of most immune infiltrating cells (Figures 8(c)–8(d)). Then, to investigate the potential clinical value of IRGS in immunotherapy, we compared the expression levels of 8 immune checkpoint-related genes (PDCD1LG2, PDCD1, PD-L1, LAG3, TIGIT, HAVCR2, IDO1, and CTLA-4) between the high-risk and low-risk groups in the TCGA cohort. We found that PDCD1LG2, PDCD1, PD-L1, LAG3, TIGIT, IDO1, and CTLA-4 were significantly upregulated in patients with low-risk scores (Supplementary Figure 3). Together, our findings suggest that IRGS can be used as a promising predictive indicator and therapeutic target for breast cancer immunotherapy.

### 3.7. The Expression of IRGS Signature Genes Is Significantly Correlated with the Sensitivity of Cancer Cells to Chemotherapy Drugs

**Figure 9:**
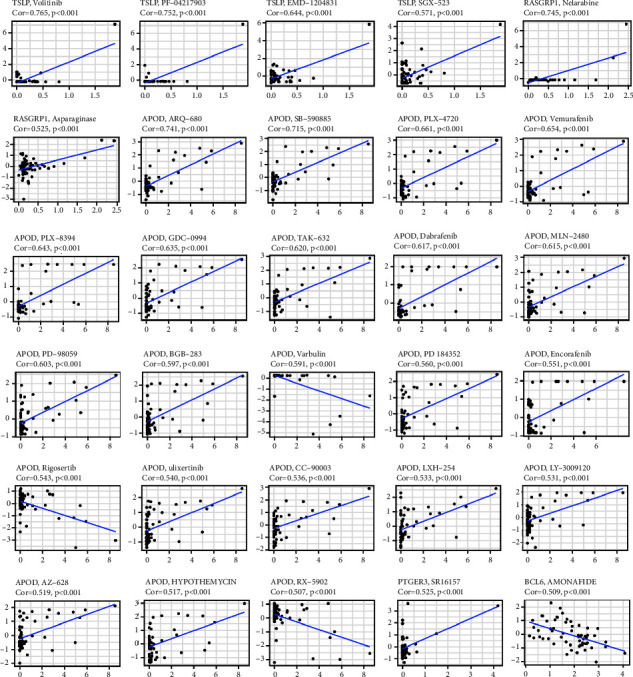
The top 30 scatter plots of Pearson correlation analysis between the expression of the signature genes and sensitivity to chemotherapeutic drugs.

Next, Pearson correlation analysis was performed to explore the relationship between the expression of the signature genes and drug sensitivity. The results showed that high expression of TSLP, RASGRP1, APOD, and PTGER3 was correlated with cancer cell drug sensitivity to a variety of chemotherapeutic drugs, especially APOD, which was highly correlated with drug sensitivity to ARQ-680, SB-590885, PLX-4720, vemurafenib, and others. However, enhanced expression of APOD was associated with increased drug resistance to Varbulin, Rigosertib, and RX-5902 in cancer cells. Similarly, high expression of BCL6 was correlated with drug resistance to amonafide (Figure 9). All results of the correlation analysis with a *P* < 0.05 were shown in Supplementary Table S4.

## 4. Discussion

In this study, we constructed and validated a novel prognostic IRGS with 11 inflammatory response genes, and this model was able to efficiently predict the prognosis of breast cancer patients. We found that the IRGS risk score was closely associated with the clinical characteristics of breast cancer patients. Besides, the IRGS risk score was able to independently predict the OS of breast cancer patients. Furthermore, we constructed a nomogram using the IRGS risk score, age, and tumor stage in the TCGA cohort to predict the 1-, 3-, and 5-year OS of breast cancer patients. It was found the IRGS risk score was correlated with tumor immune status. Finally, we found that the expression levels of the prognostic genes were significantly correlated with tumor cell sensitivity to chemotherapeutic drugs.

In recent years, accumulating evidence has indicated that inflammation mediators including cytokines, chemokines, pattern recognition receptors, activated transcriptional factors, and tumor microenvironment regulatory factors play important roles in the growth, metastasis, and prognosis of a variety of human tumors, including breast cancer [19–22]. In this study, we analyzed the public human breast cancer expression profile in the TCGA cohort and chose 243 inflammatory response-related genes for analysis. Furthermore, we used univariate Cox regression analysis, LASSO regression analysis, and multivariate Cox regression to screen and identify 11 genes (ANO6, APOD, BCL6, CXCL13, HYAL3, PTGER3, RASGRP1, SCG2, SCGB1A1, SDC1, and TSLP) to construct a prognostic risk score model. Notably, these signature genes are closely associated with the progression and prognosis of various cancers including breast cancer. A previous study demonstrated that ANO6 is associated with the metastatic potential of breast cancer [23]. Low APOD expression predicts poor prognosis in colorectal cancer [24], ovarian cancer [25], and breast cancer [26]. Furthermore, previous studies have shown that BCL6 is overexpressed in breast cancer and BCL6 expression contributes to breast cancer progression [27]. It was found that a high serum level of CXCL13 protein is a potential good prognosis indicator for hepatocellular carcinoma, but it was also found to be associated with a poor prognosis in patients with prostate cancer and breast cancer [28]. In a study based on breast cancer cell lines, HYAL3 mRNA expression was demonstrated to be associated with low invasive potential [29]. In another study, high expression of the HYAL3-v1 splice variant was found to be associated with a better prognosis in lung carcinomas [30]. Previous studies have found that the PTGER3 gene is upregulated in various breast cancer subtypes [31]. High RASGRP1 expression was demonstrated to be associated with a better prognosis in breast cancer [32]. It was found that SCG2 can predict prognosis in breast cancer [33], nonsmall cell lung cancer [34], and colorectal cancer [35]. Previous reports demonstrated that SCGB1A1 is a tumor suppressor that is downregulated in human lung cancer [36–38]. High SDC1 expression is correlated with a poor prognosis in breast cancer [39]. TSLP is a cytokine that promotes Th2-mediated immune activity and is associated with a poor prognosis in breast cancer and other epithelial cancers [40].

In both the training and validation sets, we found that patients with a high-risk score had significantly shorter OS than those with a low-risk score. Furthermore, we used IHC images from the Human Protein Atlas to determine the protein expression of genes in the IRGS in breast cancer tissues and normal tissues. Numerous studies have found that inflammatory mediators are significantly associated with a variety of clinical characteristics of breast cancer [41, 42]. We investigated the relationship between risk scores and clinical characteristics and found that the risk scores were closely related to age, PR, and HER2 status in both the training and validation sets. Further, we found that a high-risk score was associated with a worse prognosis than a low-risk score in age > 60, ER-positive, PR-negative, PR-positive, and HER2-negative subgroups in both the training and validation sets. Furthermore, multivariate Cox regression analysis and a nomogram revealed that the risk score was an independent prognostic factor that could effectively predict the survival rate of breast cancer patients. The results suggest that the risk score was closely associated with various clinicopathological features of breast cancer patients and could be used as an effective tool to predict the prognosis of breast cancer patients.

TME is well known to be closely associated with tumor cell proliferation, survival, invasion, and metastasis [43]. T cells, myeloid-derived suppressor cells (MDSCs), tumor-associated macrophages (TAMs), and dendritic cells (DCs) are components of TME and have a significant impact on breast cancer development and outcomes [4]. The inflammatory response is a vital part of the systematic immune reaction, and it is important in the early recruitment of inflammatory factors and TME formation [44]. Numerous studies have suggested that inflammatory response-related genes not only activate the immune system and participate in the inflammatory response but also play a role in TME [45]. It has been demonstrated that CXCL13 plays an important role in immune cell recruitment and adaptive immune response, suggesting that it may play multiple roles in young breast cancer patients [46]. BCL6 is a key regulator of humoral immune responses and it has been shown to affect germinal center B-cell functions and immune responses [47]. In this study, we found that genes related to adaptive immune response, immune effector process, lymphocyte-mediated immunity, and positive regulation of immune response gene signatures were enriched in low-risk patients. We also found that the IRGS risk score had a dramatically inverse correlation with the abundance of immune infiltrating cells. Immunotherapy has emerged as a new treatment modality in breast cancer, with immune checkpoint blockade (ICB) to target and block PD-1, PD-L1, and CTLA-4 approved as first-line therapy in metastatic triple-negative breast cancer [48–50]. Therefore, we examined the relationships between the risk scores and the expression levels of immune checkpoint-related genes and found that PDCD1LG2, PDCD1, PD-L1, LAG3, TIGIT, IDO1, and CTLA-4 were significantly upregulated in patients with low-risk scores in the TCGA cohort, suggesting that patients with low-risk scores may benefit from ICB treatment. Taken together, our findings suggest that genes of this IRGS are critical in regulating the immune responses to breast cancer.

Our study suggests that the IRGS risk score could be used to predict the immune status and prognosis of breast cancer patients. Because this predictive model was constructed and validated using retrospective data from the TCGA and GEO public databases, more data based on prospective studies are needed to verify its clinical applicability.

## Data Availability

The original data presented in the study are included in the article/Supplementary Material. Further inquiries can be directed to the corresponding authors.

## Supplementary Materials

Supplementary Materials Supplementary Figure 1: identification of the candidate inflammatory response-related genes in the TCGA cohort. (A) Heatmap showed 243 DEGs between breast cancer tissues and normal tissues. (B) The association between gene expression and OS was determined by univariate Cox regression analysis in the TCGA cohort. (C) LASSO coefficient profiles of the expression of 45 OS-related genes. Supplementary Figure 2: the correlation between the risk scores and clinical characteristics. Boxplot showed the correlation between the risk score and clinical characteristics in the TCGA cohort (A), and the GSE96058 cohort (B). Supplementary Figure 3: correlation between IRGS and immune checkpoints. The expression of immune checkpoints in distinct risk groups. Supplementary Table 1: genes contained in the “GOBP_INFLAMMATORY_RESPONSE” gene set. Supplementary Table 2: genes contained in the “HALLMARK_INFLAMMATORY_RESPONSE” gene set. Supplementary Table 3: genes and corresponding COEFs in the TCGA Cohort. Supplementary Table 4: chemotherapy drugs and their correlation with IRGS genes.
