# Supplementary material for: A Novel Inflammatory Response-Related Gene Signature Predicts Immune Status and Prognosis of Breast Cancer
Source: J Oncol. 2022 Nov 23;2022:5468858. doi: 10.1155/2022/5468858 (PMC9711960; doi:10.1155/2022/5468858)
Supplement: Supplementary Materials — Supplementary Figure 1: identification of the candidate inflammatory response-related genes in the TCGA cohort. (A) Heatmap showed 243 DEGs between breast cancer tissues and normal tissues. (B) The association between gene expression and OS was determined by univariate Cox regression analysis in the TCGA cohort. (C) LASSO coefficient profiles of the expression of 45 OS-related genes. Supplementary Figure 2: the correlation between the risk scores and clinical characteristics. Boxplot showed the correlation between the risk score and clinical characteristics in the TCGA cohort (A), and the GSE96058 cohort (B). Supplementary Figure 3: correlation between IRGS and immune checkpoints. The expression of immune checkpoints in distinct risk groups. Supplementary Table 1: genes contained in the “GOBP_INFLAMMATORY_RESPONSE” gene set. Supplementary Table 2: genes contained in the “HALLMARK_INFLAMMATORY_RESPONSE” gene set. Supplementary Table 3: genes and corresponding COEFs in the TCGA Cohort. Supplementary Table 4: chemotherapy drugs and their correlation with IRGS genes. [file 5468858.f1.zip › Supplementary Materials/Supplementary Figures.docx]

# Supplementary Information

**Supplementary Figures and Figure legends**


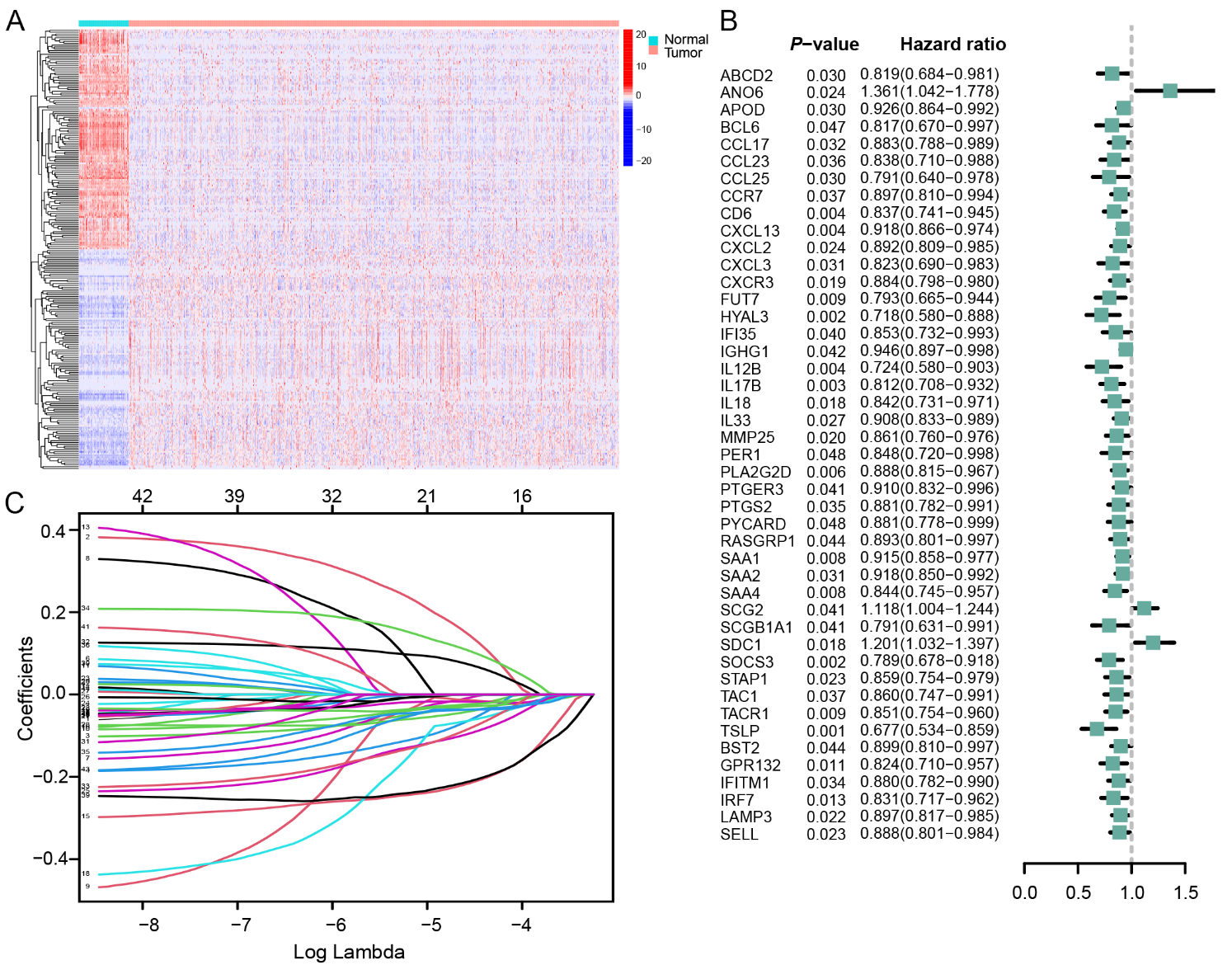


**Supplementary Figure 1** | Identification of the candidate inflammatory response-related genes in the TCGA cohort. (**A**) Heatmap showed 243 DEGs between breast cancer tissues and normal tissues. (**B**) The association between gene expression and OS was determined by univariate Cox regression analysis in the TCGA cohort. (**C**) LASSO coefficient profiles of the expression of 45 OS-related genes.


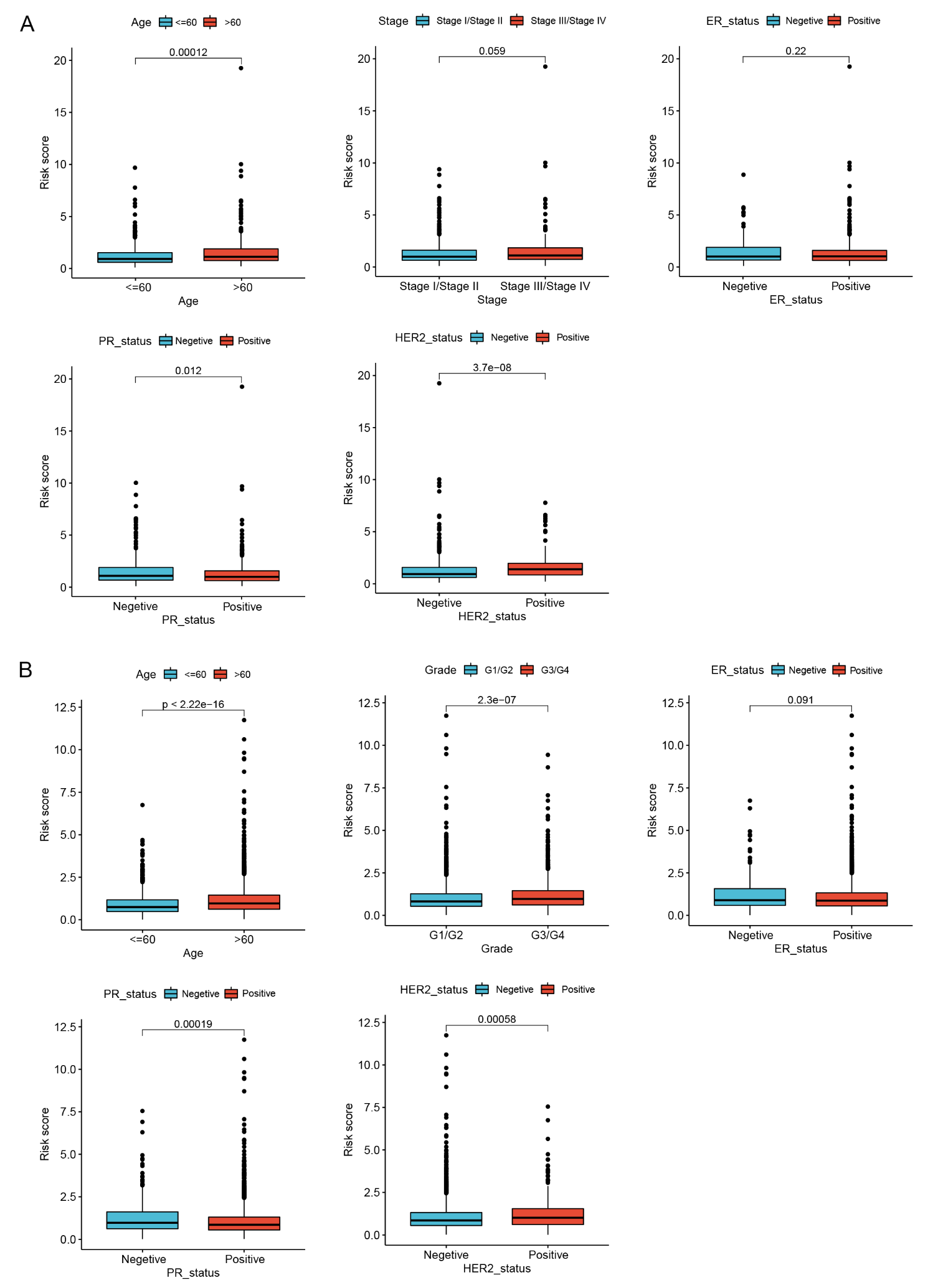


**Supplementary Figure** **2** | The correlation between the risk scores and clinical characteristics. Boxplot showed the correlation between the risk score and clinical characteristics in the TCGA cohort (**A**), and the GSE96058 cohort (**B**).


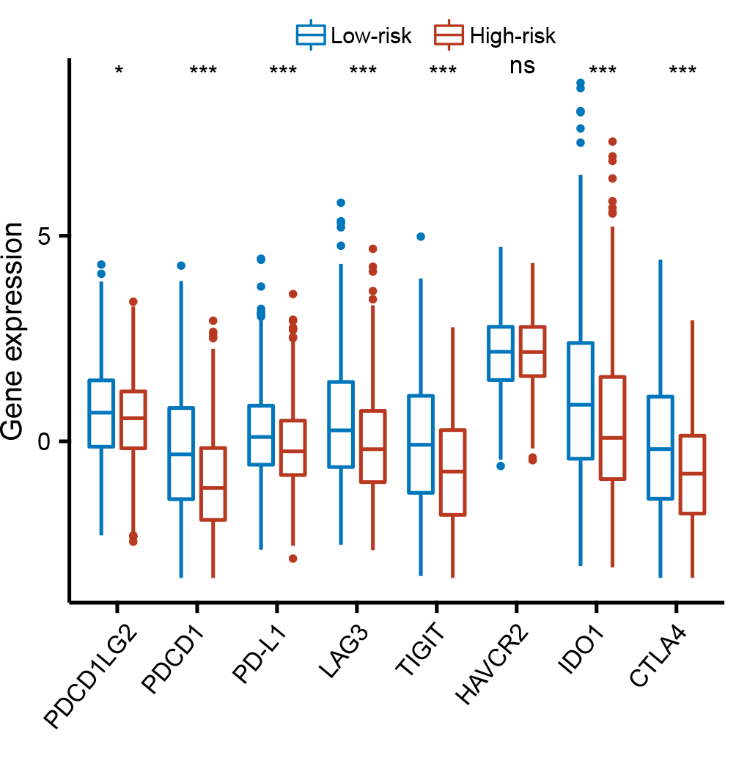


**Supplementary Figure 3** | Correlation between IRGS and immune checkpoints. The expression of immune checkpoints in distinct risk groups.
